# Long‐term antagonistic effect of increased precipitation and nitrogen addition on soil respiration in a semiarid steppe

**DOI:** 10.1002/ece3.3536

**Published:** 2017-11-09

**Authors:** Hongyan Han, Yue Du, Dafeng Hui, Lin Jiang, Mingxing Zhong, Shiqiang Wan

**Affiliations:** ^1^ International Joint Research Laboratory for Global Change Ecology School of Life Sciences Henan University Kaifeng Henan China; ^2^ College of Life Sciences University of Chinese Academy of Sciences Beijing China; ^3^ Department of Biological Sciences Tennessee State University Nashville TN USA; ^4^ School of Biology Georgia Institute of Technology Atlanta GA USA

**Keywords:** carbon cycling, climate change, grassland, long‐term dynamics, resource availability

## Abstract

Changes in water and nitrogen (N) availability due to climate change and atmospheric N deposition could have significant effects on soil respiration, a major pathway of carbon (C) loss from terrestrial ecosystems. A manipulative experiment simulating increased precipitation and atmospheric N deposition has been conducted for 9 years (2005–2013) in a semiarid grassland in Mongolian Plateau, China. Increased precipitation and N addition interactively affect soil respiration through the 9 years. The interactions demonstrated that N addition weakened the precipitation‐induced stimulation of soil respiration, whereas increased precipitation exacerbated the negative impacts of N addition. The main effects of increased precipitation and N addition treatment on soil respiration were 15.8% stimulated and 14.2% suppressed, respectively. Moreover, a declining pattern and 2‐year oscillation were observed for soil respiration response to N addition under increased precipitation. The dependence of soil respiration upon gross primary productivity and soil moisture, but not soil temperature, suggests that resources C substrate supply and water availability are more important than temperature in regulating interannual variations of soil C release in semiarid grassland ecosystems. The findings indicate that atmospheric N deposition may have the potential to mitigate soil C loss induced by increased precipitation, and highlight that long‐term and multi‐factor global change studies are critical for predicting the general patterns of terrestrial C cycling in response to global change in the future.

## 1 INTRODUCTION

Soil respiration releases 75–80 Pg CO_2_‐C per year from soil to the atmosphere, which is ~11 times of the carbon (C) released from fossil fuel combustion, and contributes significantly to global C cycling (Raich & Potter, 1995; Raich, Potter, & Bhagawati, 2002; Raich & Schlesinger, 1992). Soil respiration, constituted of autotrophic respiration and heterotrophic respiration, is generally recognized to be regulated by three fundamental driving factors (i.e., soil temperature, water availability, and C substrate). Changes in climate (e.g., climate warming and changing precipitation regime) and atmospheric composition (e.g., greenhouse gas enrichment and active nitrogen (N) deposition; Erisman, Galloway, Seitzinger, Bleeker, & Butterbach‐Bahl, 2011; Gruber & Galloway, 2008) could profoundly influence soil respiration through altering its three driving factors, with consequent feedbacks to atmospheric CO_2_ concentration and climate change (Flanagan, Sharp, & Letts, 2013; Gomez‐Casanovas, Hudiburg, Bernacchi, Parton, & DeLucia, 2016; Liu et al., 2016; Suseela, Conant, Wallenstein, & Dukes, 2012; Yan, Chen, Huang, & Lin, 2011).

Changing precipitation regimes (Zhang et al., 2007) and atmospheric N deposition (Erisman et al., 2011; Gruber & Galloway, 2008) can both affect plant growth and C uptake via altering soil water and N availability especially in ecosystems where they are limited (LeBauer & Treseder, 2008; Niu et al., 2008), with consequent impacts on autotrophic and heterotrophic soil respiration. Numerous previous studies have reported soil respiration responses to precipitation change (Flanagan et al., 2013; Liu et al., 2016; Suseela et al., 2012; Wan, Norby, Ledford, & Weltzin, 2007; Yan et al., 2011) or N addition (Chen, Li, Lan, Hu, & Bai, 2016; Graham et al., 2014; Janssens et al., 2010; Ramirez, Craine, & Fierer, 2012), but few have investigated the combined effects of these two factors (Eisenhauer, Cesarz, Koller, Worm, & Reich, 2012; Hungate, Hart, Selmants, Boyle, & Gehring, 2007; Niu et al., 2009). Meta‐analyses have revealed that the responses of plant biomass to N addition increase with mean annual precipitation (Harpole, Potts, & Suding, 2007; Xia & Wan, 2008). Effects of increased precipitation on ecosystem C exchange varied with N addition (Harpole et al., 2007; Niu et al., 2009). Considering that soil respiration consumes and is ultimately under the control of C substrate from ecosystem productivity (Wertin, Belnap, & Reed, 2017; Yan et al., 2011) due to the biochemical processes of plant roots and soil microorganisms, it is reasonable to predict that precipitation change and N addition could also interactively impact soil respiration.

Biological components including plants and soil microorganisms in natural ecosystems have well adapted to environmental fluctuations during their evolutionary history. When facing abrupt changes of global change driving factors, especially in manipulative experiments (Klironomos et al., 2005; Luo & Reynolds, 1999), plants and soil microorganisms may adjust the physiological activities, leading to different short‐ and long‐term response patterns of soil respiration to climate change (Melillo et al., 2002; Nielsen & Ball, 2015). Moreover, changes in plant community composition and soil nutrient status due to long‐term study duration would also alter effects of experimental treatment on ecosystem functions (Fry et al., 2013; LeBauer & Treseder, 2008). Thus, findings of the short‐term studies (Flanagan et al., 2013; Suseela et al., 2012; Yan et al., 2011) may not be able to predict long‐term responses of soil respiration as effects of experimental treatments do not always remain consistent over different temporal scales (Blankinship, Niklaus, & Hungate, 2011; Tilman, Reich, & Isbell, 2012; Zhou et al., 2014). For example, a previous study has reported that the positive effects of increased precipitation on soil respiration could be intensified with time in a semiarid grassland during a 3‐year field experiment (Liu, Zhang, & Wan, 2009). Positive impacts of N enrichment on soil microbes are likely observed at experimental sites with shorter durations (Treseder, 2008), but may disappear over a long term (Bowden, Davidson, Savage, Arabia, & Steudler, 2004). Moreover, the temporal variations of soil respiration to N addition are often observed under ambient precipitation (Chen et al., 2016; Zhou et al., 2014). It is still unknown whether the long‐term response pattern of soil respiration to N addition would change under different precipitation scenarios.

A 9‐year field manipulative experiment with increased precipitation and N addition has been conducted since 2005 in a semiarid grassland in northern China where precipitation and N are both limited (Liu et al., 2009; Niu et al., 2008). Based on the previous studies, the following questions were addressed: (1) did increased precipitation and N addition interactively impact on soil respiration; (2) and did effects of increased precipitation or N addition on soil respiration change at different temporal scales.

## 2 MATERIALS AND METHODS

### 2.1 Site description

This study was part of Duolun Global Change Multiple‐factor Experiment (GCME‐Duolun) located in a temperate steppe in Duolun County (42°11′N, 116°48′E, 1324 m a.s.l) of the Inner Mongolia Autonomous Region, China. The long‐term (1960–2013) mean annual precipitation (MAP) in the local area is 374.5 mm, with ~90% distributed between May and October. Mean monthly temperature varies from −17.3°C in January to 19.1°C in July, and mean annual air temperature (MAT) is 2.4°C (China Meteorological Administration). The soil type is Haplic Calcisols according to FAO classification, or chestnut in Chinese classification. Soil in the study area is constituted of 16.95% clay, 20.30% silt, and 62.75% sand (Niu et al., 2008).Vegetation of the typical temperate steppe is dominated by *Artemisia frigida*,* Leymus chinensis*,* Stipa krylovii*,* Potentilla acaulis*,* Cleistogenes squarrosa*,* Agropyron cristatum*, and *Allium bidentatum* with average coverage of approximately 30%, 10%, 7%, 5%, 4%, 2%, and 1%, respectively in 2013. For more details of plant species see Yang et al. (2011) in the same study site. The growing season lasts from May to October.

### 2.2 Experimental design

**Figure 1:**
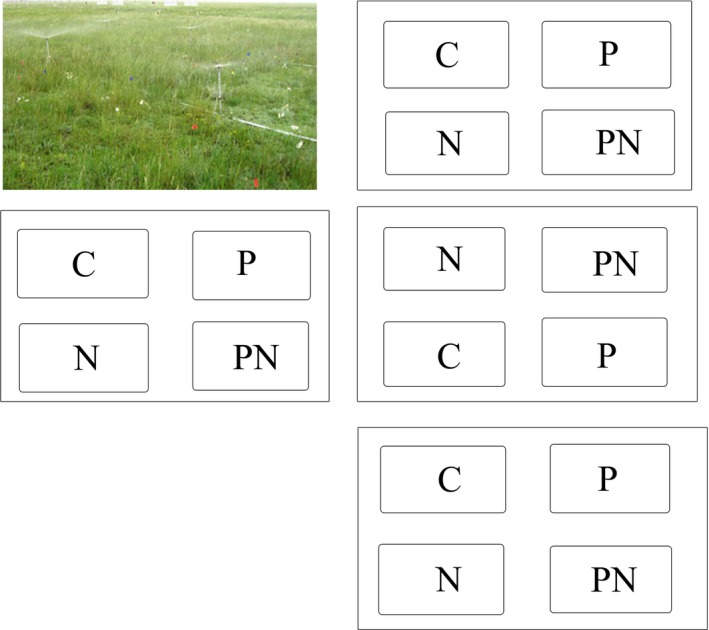
Site layout of the study

The GCME‐Duolun experiment (http://gce.henu.edu.cn/english/EXP3-GCME-Duolun.htm) was initiated in April 2005 and included four factors: mowing, N and phosphorus addition, increased precipitation, and warming. This study was part of GCME‐Duolun experiment, which employed a factorial design with four blocks (Figure 1). There were four treatments in each block (see Niu et al., 2009 for detailed information). The four treatments included control (C), increased precipitation (P), N addition (N), and increased precipitation plus N addition (PN). The total number of plots was 16 (Figure 1). Moreover, there were another 16 plots with the same treatments (i.e., C, P, N, and PN) adjacent to the study site, except that the 16 plots were assigned to four blocks with mowing at the end of August each year. In the increased precipitation plots, there were six sprinklers which were evenly distributed in two rows (image in the top left corner of Figure 1). The six sprinklers covered the whole increased precipitation plots. Fifteen millimeter water was added weekly in July and August each year under the increased precipitation treatment. The total amount of precipitation added was 120 mm each year, which was approximately 30% of mean annual precipitation in this area. Nitrogen was added in the form of urea (10 g N m^−2^ year^−1^) once a year in the middle of July. The N addition level was close to the maximum deposition rate in northern China (He, Liu, Fangmeier, & Zhang, 2007).

### 2.3 Soil respiration, soil temperature, and soil moisture

A PVC collar (11 cm in diameter and 5 cm in height) was permanently inserted 2.8 cm into soil in each plot. Soil respiration in the growing season was measured twice a month from 2005 to 2010, and three times a month from 2011 to 2013 with a Li‐8100 portable soil CO_2_ fluxes system (Li‐Cor Inc., Lincoln, NE, USA). Soil respiration measurements were performed between 9:00 a.m. and 12:00 a.m. to eliminate diurnal variations. Aboveground parts of living plants inside the PVC collars were removed 2–3 days before soil respiration measurements and were left inside the collars to decompose. Furthermore, if there was a rainfall event, the measurement was carried out 2–3 days later to reduce the pulse effects of precipitation on soil respiration.

Soil temperature at the depth of 10 cm was measured at the same time with the measurement of soil respiration using a thermocouple probe (Li‐8100‐201) attached to the Li‐8100. Soil moisture (% m^3^/m^3^) of 0–10 cm was measured with a Diviner‐2000 Portable Soil Moisture Probe (Sentek Pty Ltd., Balmain, Australia) twice a month in 2005–2007, and 3–6 times a month in 2008–2013.

### 2.4 Gross ecosystem productivity

Gross ecosystem productivity (GEP) was calculated as follows:

GEP=ER−NEE

where NEE is net ecosystem C exchange, and ER is ecosystem respiration. Both NEE and ER were measured with a transparent chamber (0.5 × 0.5 × 0.5 m^3^) attached to an infrared gas analyzer (IRGA; Li‐6400; Li‐Cor, Lincoln, NE, USA). During the measurements of NEE, the chamber was put on the aluminum frame inserted in each plot to record the CO_2_ concentrations. ER was measured using the same method except that the chamber was covered with an opaque cloth. For more information about the measurement of NEE and ER see Xia, Niu, and Wan (2009).

### 2.5 Root biomass

Three 40‐cm‐deep hole was excavated with a soil auger (5‐cm internal diameter) in each plot at the end of August since 2009. Soil was sieved through a 2‐mm screen, and roots were washed and dried (65°C for 48 hr) to measure the root biomass. Root biomass was expressed as the weight in unit area in one growing season.

### 2.6 Vegetation sampling

There was one permanent quadrat (1 × 1 m) in each plot. A frame equally separated into 100 grids (10 × 10 cm) was put above the quadrat to investigate the structure of plant community in August every year when plant biomass was at the peak. For more details of vegetation measurements see Yang et al. (2011). Species diversity (*D*; Romme, 1982) was calculated using the modified Simpson index expressed as the following equation:

$$D=-\ln\left[\sum {\left(N_{i}/N\right)}^{2}\right]$$

where *N*_*i*_ is the number of individuals of plant species *i*, and *N* is the total number of plants species in each quadrant.

### 2.7 Data analysis

The growing‐season averages of each index were calculated from the monthly mean values. The monthly means of each growing season and the seasonal means of soil respiration, temperature, and moisture were tested using a mixed‐effects model with repeated measurements (Proc Mixed, SAS 8.1; SAS Institute Inc., Cary, NC, USA). Precipitation and nitrogen treatments were fixed factors, and plots were assigned as random factors. First‐order autoregressive structure (AR (1)) was chosen as the covariance structure according to akaike information criterion (AIC). Effects of increased precipitation on soil respiration were calculated as [100 × (P − Control)/Control] without N addition and [100 × (PN − N)/N] with N addition. N effects were calculated as [100 × (N − Control)/Control] in ambient precipitation plots and [100 × (PN − P)/P] under increased precipitation. Precipitation and N effects were calculated using the seasonal mean values. Simple regression analysis and stepwise multiple linear analyses were used to test the relationships of soil respiration with soil temperature, soil moisture, and GEP. All statistical analyses were conducted using SAS V.8.1 software (SAS Institute Inc., Cary, NC, USA).

## 3 RESULTS

### 3.1 Soil temperature and soil moisture

**Figure 2:**
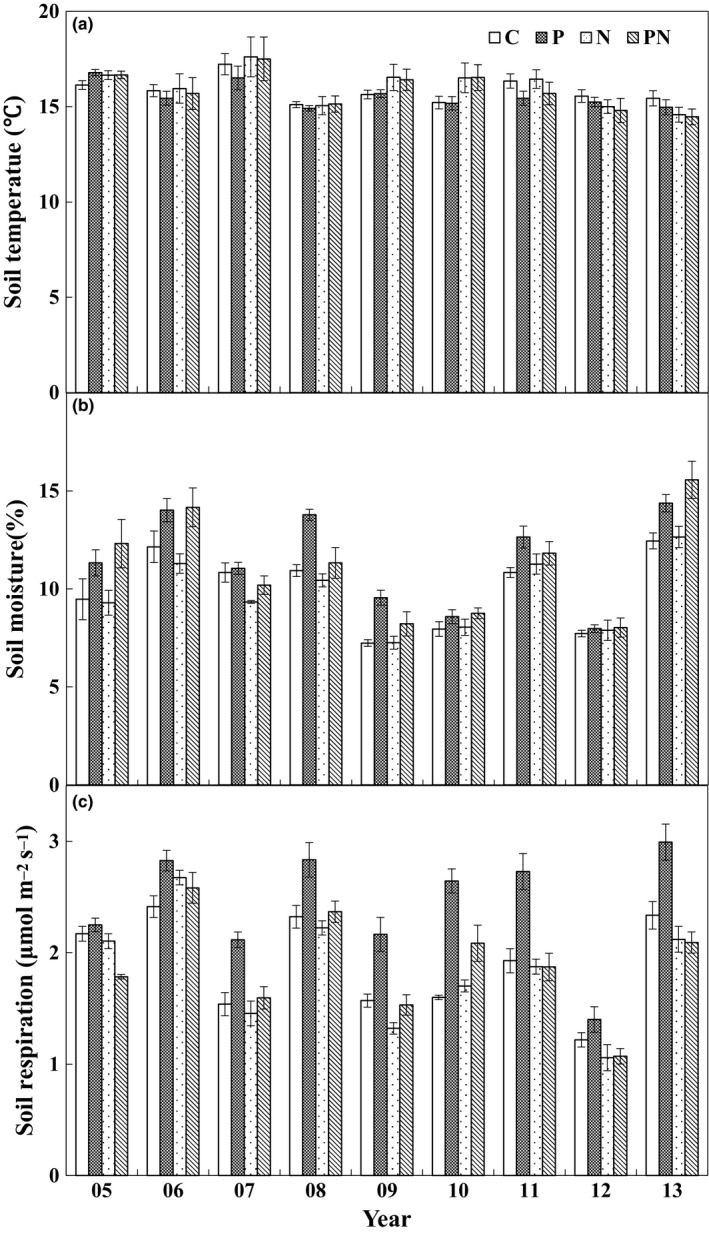
Growing‐season mean soil temperature (a), soil moisture (b), and soil respiration (c) under different treatments over the 9 years. C, the control; P, the increased precipitation; N, the nitrogen addition; PN, the increased precipitation plus nitrogen addition. Error bars indicate ± *SE* (*n* = 4)

During the whole experimental period from 2005 to 2013, growing‐season mean soil temperature (15.8°C) ranged from 15.1 to 17.2°C in the control plots (Figure 2a). Increased precipitation, N addition, or their interactions did not affect soil temperature (all *p *>* *.05, Table 1). Growing‐season mean soil moisture varied over years (*p *<* *.001, Table 1), ranging from 7.24% in 2009 to 12.45% in 2013 in the control plots with an average value of 9.96% across the 9 years (Figure 2b). Increased precipitation stimulated soil moisture by 1.48% (absolute change). Neither N addition nor its interaction with increased precipitation affected soil moisture (Table 1). However, the effects of increased precipitation changed with year (*p *<* *.001, Table 1). Increased precipitation stimulated soil moisture over the 9 years (*p *<* *.05) except that in 2007, 2010, and 2012.

**Table 1:** Results (*F*‐values) of repeated measures ANOVAs on the effects of increased precipitation (P), nitrogen addition (N), year (yr), and their potential interactions on soil temperature (ST, °C), soil moisture (SM, %m^3^/m^3^), and soil respiration (SR, μmol m^−2^ s^−1^) from 2005 to 2013

Source of variation	*df*	ST	SM	SR
Block	3	14.35***	1.35	3.34*
P	1	1.37	18.74***	43.85***
N	1	2.03	0.67	48.10***
P*N	1	0.08	0.02	30.55***
Yr	8	22.29***	125.09***	150.61***
P*yr	8	0.97	4.60***	5.95***
N*yr	8	2.60*	1.54	4.00***
P*N*yr	8	0.25	1.39	1.60

Significant level: *p <* *.05, **p <* *.01, ***p <* *.001.

### 3.2 Effects of increased precipitation and N addition on soil respiration

There were strong interannual variations of soil respiration during the 9‐year experimental period (*F *=* *150.6, *p *<* *.001), with seasonal mean soil respiration in the control plots ranging from 1.22 μmol m^−2^ s^−1^ in 2012 to 2.41 μmol m^−2^ s^−1^ in 2006 (Figure 2c). Increased precipitation, N addition, and their interactions had significant influences on soil respiration over the 9 years (all *p *<* *.001, Table 1). Mean soil respiration was enhanced by 15.8% under increased precipitation. By contrast, N addition suppressed soil respiration by 14.2%. Increased precipitation stimulated soil respiration by 28.4% without N addition and by 2.7% with N addition, whereas N addition reduced soil respiration by 3.3% and 22.7% in the ambient and elevated precipitation plots, respectively. The main effects of increased precipitation and N addition on soil respiration varied with year (all *p *<* *.01, Table 1). Increased precipitation stimulated soil respiration by 24.0%, 14.4%, 27.8%, 43.3%, 21.0%, and 14.1% in 2007, 2008, 2009, 2010, 2011, and 2013, respectively, marginally decreased it in 2005 (*p *<* *.10), but had no impacts in 2006 and 2012 (both *p *>* *.10, Figure 2c, Table 2). N addition reduced soil respiration in eight of the 9 years except for the year of 2006 (Figure 2c, Table 2).

**Table 2:** Results (*F*‐values) of repeated measures of ANOVAs on the effects of increased precipitation (P), nitrogen addition (N), and their potential interactions on soil respiration in each growing season

Season	Block	P	N	P*N
DF	3	1	1	1
2005	8.07**	6.79^	32.87*	18.30*
2006	5.69*	2.20	0.01	5.39*
2007	17.54***	12.34**	8.75*	4.59^
2008	0.15	8.65*	6.46*	2.69
2009	5.98*	15.27**	18.31**	3.44^
2010	11.11**	44.80***	4.56^	9.61**
2011	7.78**	17.49**	22.88***	17.79**
2012	9.40**	1.11	7.02*	0.87
2013	5.20*	5.52*	17.43**	6.58*

Significant level: ^*p *<* *.10, *p <* *.05, **p <* *.01, ***p <* *.001.

**Figure 3:**
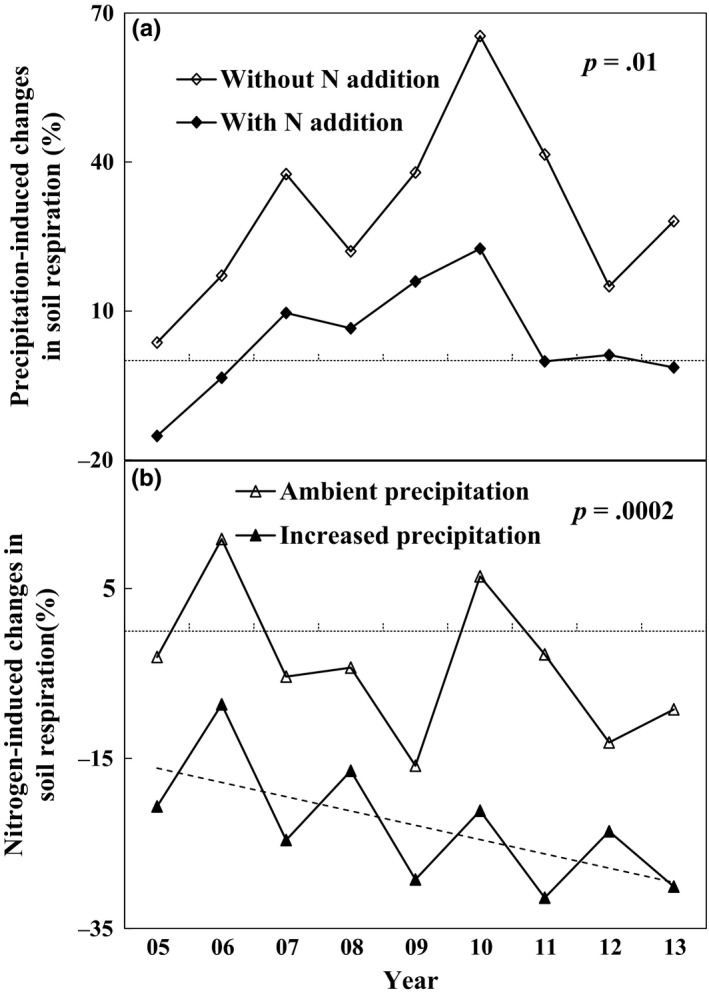
The percentage change of soil respiration induced by the increased precipitation (a) and the nitrogen addition (b) from 2005 to 2013. *p*‐value in the figure represents the significant test of difference of precipitation effects between N addition and without N addition, or the difference of N effects between ambient and increased precipitation treatments

Effects of increased precipitation on soil respiration depended on N addition in seven of 9 years (Table 2). The precipitation‐induced changes in soil respiration were substantially lower or even became negative with N addition (Figure 3a). Under the ambient precipitation, N addition generally decreased soil respiration across the 9 years except in 2006 and 2010. Increased precipitation amplified the negative responses of soil respiration to N addition during the whole experimental period (Figure 3b).

### 3.3 Long‐term effects of increased precipitation and N addition on soil respiration

**Figure 4:**
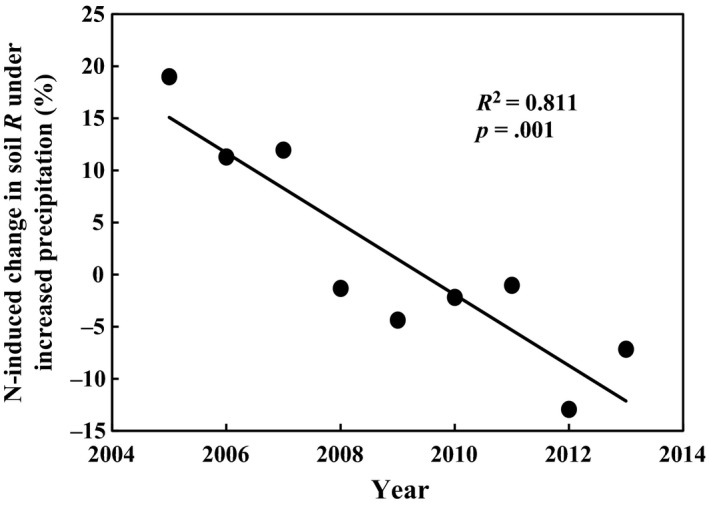
Nitrogen‐induced change of soil respiration (R) under increased precipitation in the plots with mowing

**Figure 5:**
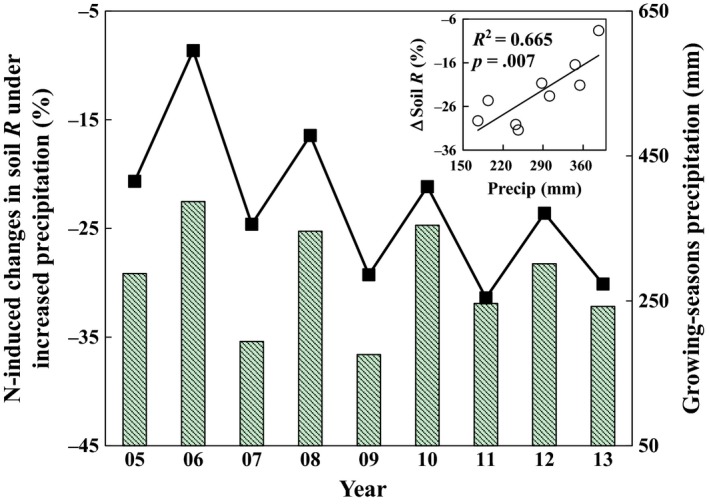
The nitrogen‐induced change in soil respiration (R, %) under the increased precipitation (line) and growing‐season precipitation amounts (mm, bars) from 2005 to 2013. The linear relationship is shown in the inset

During the 9‐year experimental period, the precipitation‐induced changes in soil respiration showed larger interannual variability than the N‐induced changes in soil respiration (Figure 3). Nitrogen addition‐induced changes in soil respiration showed a declined trend with time under the increased precipitation (*R* ^2^ = 0.40, *p *=* *.067, Figure 3b), but not under the ambient precipitation. The declined pattern of N addition‐induced changes under the increased precipitation was also observed in another study site with mowing (*R* ^2^ = 0.81, *p *=* *.001, Figure 4). The responses of soil respiration to N addition showed a 2‐year oscillation during the whole experimental period under the increased precipitation (Figures 3b and 5). A similar 2‐year oscillation was also observed for the growing‐season (May to October) precipitation amount from 2005 to 2013 (Figure 5). The two oscillation patterns were synchronous and positively correlated with each other (*R* ^2^ = 0.665, *p *=* *.007; Figure 5 inset).

### 3.4 Control factors over the interannual variability of soil respiration

**Figure 6:**
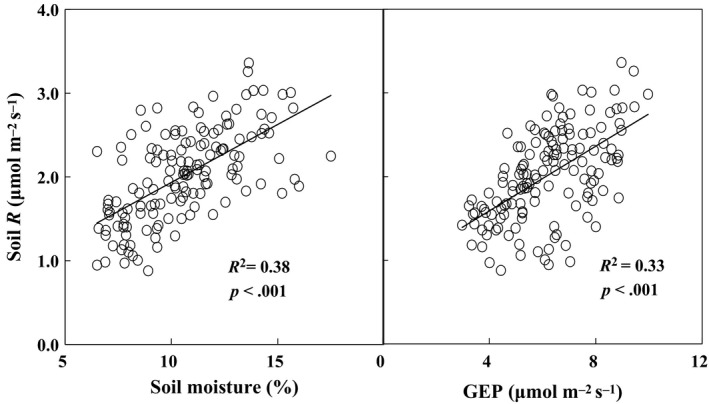
Relationships of soil respiration with soil moisture (a) and gross ecosystem productivity (GEP, b). Each data point represents growing‐season mean in each of the 16 plots from 2005 to 2013

**Figure 7:**
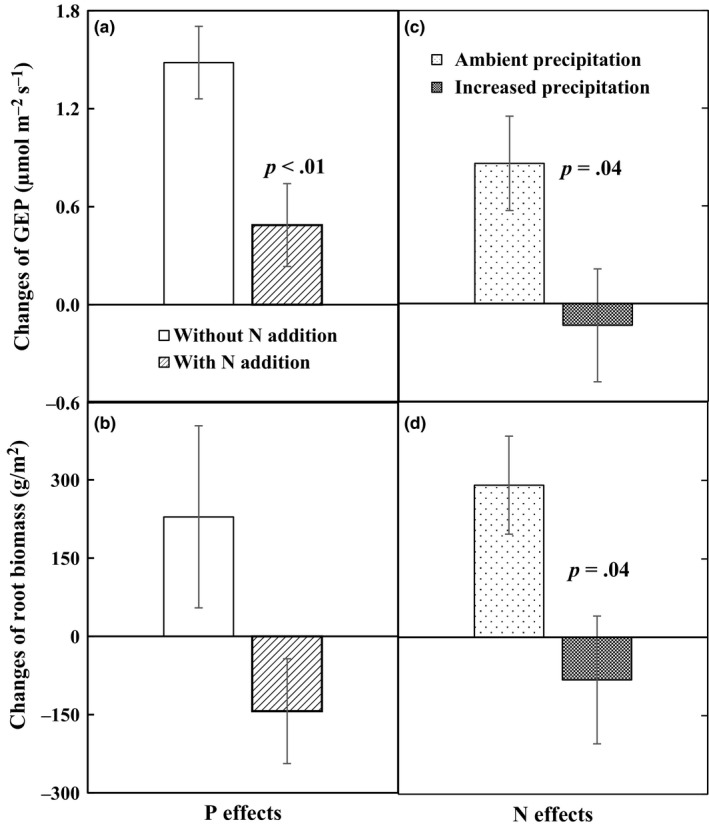
Effects of increased precipitation on gross ecosystem productivity (GEP, a) and belowground biomass (b) in plots without and with nitrogen addition, and effects of nitrogen addition on gross ecosystem productivity (GEP, c) and root biomass (d) in ambient and increased precipitation plots. *p*‐values represent the significant difference between the two bars

**Figure 8:**
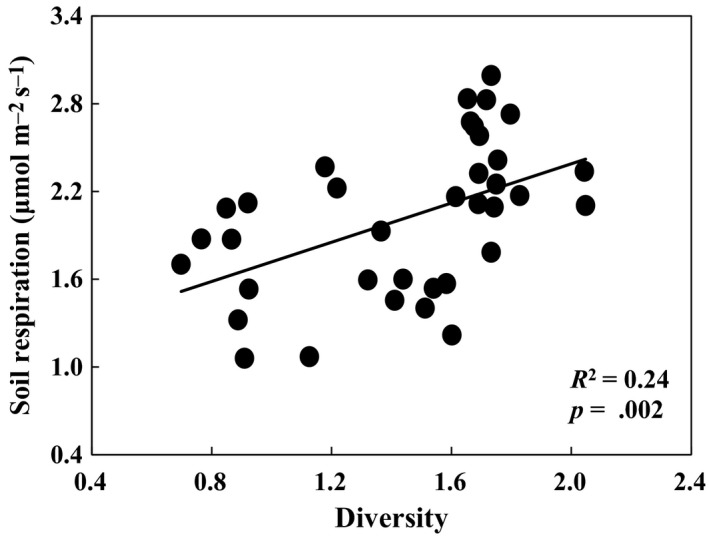
Relationship of soil respiration with plant species diversity. Each data point represents the average in each of the four treatments from 2005 to 2013

**Figure 9:**
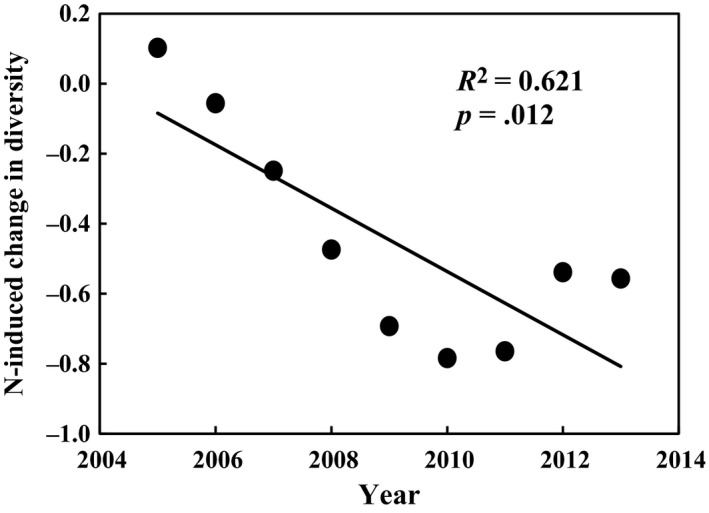
Effect of nitrogen addition on plant species diversity changed with experimental duration

Three main explanatory factors (i.e., soil temperature, soil moisture, and GEP) were used to explain the interannual variability of soil respiration in the long‐term experiment. Although soil respiration increased exponentially with soil temperature within each growing season (all *p *<* *.05), growing‐season mean soil respiration did not show significant relationships with growing‐season mean soil temperature over the 9 years. Results of linear regression model demonstrated that soil moisture and GEP contributed 38% and 33% of the year‐to‐year variability of soil respiration, respectively (Figure 6). The stepwise multiple linear analyses revealed that the combination of soil moisture and GEP explained 50% variations of soil respiration (*p *<* *.0001). In addition, changes of GEP induced by increased precipitation in plots without N addition were larger than plots with N addition. N addition increased GEP in plots with ambient precipitation, but reduced it in plots with increased precipitation (Figure 7a,c), resulting in no effects of N addition on GEP. Root biomass, as component of GEP, showed similar pattern in response to increased precipitation and N addition. Nitrogen addition suppressed the positive effects of increased precipitation on root biomass, and even turn it into negative effects (Figure 7b). Increased precipitation also changed the effect of N addition on root biomass from positive to negative effects (Figure 7d). In addition, soil respiration was positively correlated with plant species diversity (Figure 8) which showed a declining pattern under N addition treatment over the 9 years (*R* ^2^ = 0.621, *p *=* *.012; Figure 9).

## 4 DISCUSSION

The 9‐year manipulated study has examined responses of soil respiration to increased precipitation and N addition, and their interactive effects. In this study, soil respiration was interactively influenced by the positive effect of increased precipitation and negative effects of N addition. Furthermore, effects of precipitation and N on soil respiration changed with different temporal scales. The interannual variations of soil respiration were positively correlated with explanatory factors, such as soil moisture, gross ecosystem productivity (GEP), plant species diversity.

### 4.1 Antagonistic effects of increased precipitation and N on soil respiration

Although the antagonistic effect of increased precipitation and N addition on soil respiration (Figure 2) has not been reported before, a similar response pattern of gross ecosystem productivity (GEP) was observed for the first 4 years (2005–2008) in this same experiment (Niu et al., 2009), but not for plant biomass in a mesotrophic grassland ecosystem (Lee, Manning, Walker, & Power, 2014). Given that soil respiration is substrate‐regulated processes and GEP can provide C substrate for root and soil microbial respiration (Flanagan et al., 2013; Yan et al., 2011). Plant photosynthesis, growth and productivity represented by GEP can regulate soil respiration and its response to environmental change (Bai et al., 2010; Tang, Misson, Gershenson, Cheng, & Goldstein, 2005; Wertin et al., 2017). The positive dependence of soil respiration upon GEP (Figure 6) supported the above argument. In addition, the smaller precipitation‐induced enhancement of GEP with N addition than without N addition over the 9 years could largely explain the lower precipitation effect on soil respiration under N enrichment (Figure 7a). Similarly, N addition stimulated GEP under ambient precipitation, but decreased it under increased precipitation over the 9 years (Figure 7c), which could partly contribute to the exacerbated negative N responses of soil respiration under the increased precipitation scenarios.

Furthermore, enhanced soil water availability can contribute to higher diffusion rate and provide microbes and its extracellular enzyme to have more access to gain C substrates for respiration (Davidson & Janssens, 2006; Manzoni, Schimel, & Porporato, 2012), especially in water‐limited ecosystem. Therefore, increased precipitation in the semiarid grassland stimulates soil respiration due to the enhanced soil water availability (Wu, Dijkstra, Koch, PeÑUelas, & Hungate, 2011). However, soil water availability is not altered under N addition, as plant community could balance the evaporation and transpiration to maintain a relatively constant evapotranspiration (Tian et al., 2016). Increased precipitation and N addition have no interaction on soil water content, but the two resources do interactively affect soil respiration. Therefore, although soil respiration positively depends on soil water availability, different changes on soil water content might not be the reason to explain the interaction on soil respiration.

Plant species diversity, as an integrated index, could indirectly affect soil respiration due to changes in plant and microbial community composition and structure which would therefore affect the three main control factors (i.e., soil temperature, soil moisture, and GEP) and the activity of soil microorganisms (Compton, Watrud, Porteous, & DeGrood, 2004; Kardol, Cregger, Campany, & Classen, 2010; Steinauer et al., 2015). Therefore, reduction in plant diversity (Yang et al., 2011) in combination with no response of soil moisture and GEP under N addition would have resulted in the reduction of soil respiration. In addition, soil pH has been reported to be reduced by N addition in the same study ecosystem of GCME‐Duolun experiment (Chen et al., 2014). As pH could suppress soil microbial community structure and activities (Bowden et al., 2004), the decreased soil pH could also partly contribute to the negative effects of N addition on soil respiration. Previous study has reported that increased soil water content could stimulate soil N availability (Borken & Matzner, 2009; Giese, Gao, Lin, & Brueck, 2011; Manzoni et al., 2012). Given that N enrichment has negative effect on soil respiration, the stimulation of N availability induced by increased precipitation could further intensify the negative impacts of N addition on soil respiration. Moreover, with the mitigation of limited resources, such as soil water and N availability, belowground C allocation from GEP would be reduced as less C substrate was needed for plant root and soil microorganisms to obtain water or N from soil (Moorhead & Sinsabaugh, 2006; Suseela & Dukes, 2013). The assumption is supported by the decreased and negative responses of root biomass induced by increased precipitation in N addition plots, and induced by N addition in increased precipitation plots in this study (Figure 7b,d). Considering that respiration of root and associated rhizosphere microbes constitutes large proportion to total soil respiration, the interactive effects of increased precipitation and N addition on root biomass could also explain the antagonistic responses of the two resources.

### 4.2 The declining pattern of N‐induced change in soil respiration

A meta‐analysis has reported a declining trend of N effects on soil respiration with experimental duration in forest ecosystems, but not in grassland ecosystems which might be eliminated by the management practice, for example, mowing or grazing (Zhou et al., 2014). However, results in our study were not consistent with the assumption from the meta‐analysis in grassland ecosystems. As mowing was also considered a treatment factor in GCME‐Duolun, another 16 plots with the same treatments (i.e., C, P, N, and PN) in this study were mowed at the end of August once a year. Plant aboveground parts were removed out of plots with the mowing treatment. In the mown plots, the exacerbated negative effect of N addition under increased precipitation did not disappear (*R* ^2^ = 0.811, *p *<* *.001, Figure 4), indicating that mowing might not be able to eliminate the decreasing trend of N effect. Further studies are needed to better understand mechanisms of mowing effects on soil respiration as mowing also plays important roles on ecosystem functions in grassland ecosystems.

Plant species diversity could positively impact soil respiration (*p *=* *.002; Figure 8) by enhancing the quantity of C substrate due to increase in ecosystem productivity (Tilman et al., 2012) and by enhancing the biomass and activity of soil microorganisms and nematodes (Steinauer et al., 2015) due to the various and high quality of C substrate in high‐diversity plant community. Therefore, the N‐induced decrease in plant species diversity (Figure 9) could contribute to the declining pattern of soil respiration with increasing duration of N addition treatment. In addition to the management practice and plant species diversity, the cumulative changes in microbial community composition (Frey, Knorr, Parrent, & Simpson, 2004) and soil acidification (Chen et al., 2014, 2016; Liu et al., 2014) induced by N addition treatment could also account for the temporal variation of soil respiration responses to N addition.

### 4.3 Long‐term oscillations of N effect on soil respiration under increased precipitation

Oscillations of the response of plant production or biomass to climate change have been reported with experimental duration (Haddad, Tilman, & Knops, 2002; Tilman & Wedin, 1991). For example, Haddad et al. (2002) observed oscillations of plant biomass persisting for 9 years which was probably caused by litter dynamics, not by the annual variation of precipitation. However, in our study, the interannual variation of precipitation in the growing season from 2005 to 2013 synchronized with the oscillation pattern of N effects on soil respiration under the increased precipitation treatment (Figure 3), which is supported by the significant relationship between soil respiration change and precipitation (*R* ^2^ = 0.665, *p *=* *.007). Therefore, interannual precipitation variability could partly explain the two‐year oscillations on soil respiration responses to N enrichment under elevated precipitation in this study.

Moreover, other two possible reasons could help explain the above oscillation pattern. First, litters play important roles in regulating plant growth, C substrate supply, and soil respiration changes in grasslands (Haddad et al., 2002; Wang et al., 2011). Greater plant growth and litter production in the year with higher precipitation often leads to light limitation which could suppress plant growth, litter production, and C substrate supply for plant roots and soil microorganism in the next year (Tilman & Wedin, 1991). This could affect interannual variations of soil respiration and its response to N addition. Second, greater litter accumulation in the previous high‐precipitation year may lead to enhanced litter decomposition and N release in the next year, especially under the N addition plus elevated precipitation treatment, which could further exacerbate the negative impacts of soil N enrichment on soil respiration (see the first section of Discussion). Unfortunately, no data on litter production from 2005 to 2013 were available to examine this argument directly in this study. However, the effect of litter could be examined indirectly using data from the mowed plots in the GCME‐Duolun where most parts of aboveground plant biomass were removed out of the plots at the end of August of each growing season. In the mown treatment, N effects on soil respiration in the increased precipitation plots did not show oscillations during the whole experimental period (Figure 4), which supported the above argument that litter production dynamics might be an important factor in controlling the 2‐year oscillations of soil respiration response. Nevertheless, further study is needed to find out whether the 2‐year oscillations would continue or not in the future.

In conclusion, using a 9‐year long‐term field experiment, we found antagonistic impacts of increased precipitation and N availability on soil respiration. The antagonistic effects of precipitation and N addition could be caused primarily by the interaction on C substrate supply from GEP and on belowground C allocation. The effects of increased precipitation on soil respiration depend on N addition, indicating that N deposition might mitigate soil CO_2_ emission due to the increased precipitation in semiarid grasslands. The N addition‐induced declining and 2‐year oscillation patterns of soil respiration under elevated precipitation during the whole experimental period could be probably caused by interannual variability of precipitation and dynamics of litter. The findings highlight the importance of long‐term multi‐factor studies in projecting terrestrial C cycling in response to climate change in the future.

## CONFLICT OF INTEREST

None declared.

## AUTHOR CONTRIBUTIONS

Shiqiang Wan conceived the ideas and designed methodology; Hongyan Han, Yue Du, and Mingxing Zhong collected the data; Hongyan Han and Shiqiang Wan analyzed the data; Hongyan Han, Shiqiang Wan, Dafeng Hui, and Ling Jiang led the writing of the manuscript. All authors contributed critically to the drafts and gave final approval for publication.
